# A Cross-Sectional Study to Evaluate Work-Life Balance Among Indian Medical Students

**DOI:** 10.7759/cureus.55293

**Published:** 2024-02-29

**Authors:** Maneeth Mylavarapu, Vaishnavi K, Umme Aiman, Kapil Usgaokar, Triasha Dutta, Sampoorna Monica Nakirakanti

**Affiliations:** 1 Public Health, Adelphi University, Garden City, USA; 2 Internal Medicine, Sapthagiri Institute of Medical Science and Research Center, Bengaluru, IND; 3 Internal Medicine, Bhaskar Medical College, Hyderabad, IND; 4 Medicine, Sussex Partnership NHS Foundation Trust, Worthing, GBR; 5 Internal Medicine, Padmashree Dr. D.Y. Patil School of Medicine, Pune, IND; 6 Internal Medicine, Kamineni Institute of Medical Sciences, Narketpally, IND

**Keywords:** spss, medical students, study-life balance, self-care, work-life balance

## Abstract

Introduction

The concept of work-life balance is a complex, multidimensional intertwinement of the roles an individual plays in their professional and personal life. Work-life balance is crucial for every profession, and doctors have no exemption not exempted from it. Medical students and young graduates face numerous challenges that potentially impact their work (study)-life balance.

Objectives of the study

The aim is to assess the hours spent in study and the hours spent in non-study activities by medical students and graduates in India and to assess the study-life balance among them.

Methodology

A cross-sectional observational study employing a predefined web-based* *survey to investigate the study-life balance among medical students and graduates across India. A predesigned questionnaire was designed and made accessible through Google Forms, which was distributed among doctors across India via popular social media platforms. Data management was conducted using Microsoft Excel and Data analysis was done using SPSS (IBM Corp., Armonk, NY).

Results

A total of 416 responses were included in the study. The study participants were predominantly female (64.2%). Most of the study participants were from the State of Telangana (63.9%). The time spent studying was < 10 hours/week for 43.8% students and 10-25 hours/week for 27.2% students. Around 24% students reported spending 10-25 hours/week in hospital. While 47.4% reported spending less than one to two hours per day with their family, 26% of the participants answered “yes” to the question “Do you feel that your study-life is stressful?.”

Conclusions

Self-care and study-life balance is a multi-factorial focal area that is based on balancing stress and happiness, with completing the tasks of the medical school. Medical students need to receive proper guidelines to transition into medical school for better study-life balance.

## Introduction

Work-life balance functions as a key contributor to a person’s well-being personally and professionally and also plays a significant role in their career decisions. Defining work-life balance is a complex task and has multiple definitions. Work-life balance can be described by categorizing into two key dimensions, namely (A) role engagement in multiple roles in work and nonwork life and (B) minimal conflict between work and nonwork roles. Each of the two key dimensions has multiple definitions. The first key dimension has four well-known definitions while the second has three [1].

The first definition (A) of work-life balance for the first dimension involves attentive engagement in multiple roles [2]. The second definition is based on equal time and involvement across multiple roles. The third talks about balanced satisfaction across all life domains, and the final (fourth) involves balanced involvement across life domains [3]. The second dimension (B) “minimal conflict between work and nonwork roles" has three definitions. The first states minimizing the role conflict between work and family [4], the second involves role enrichment with no role conflict [5], and the third reflects the best possible resource management to minimize role conflict [6]. All these definitions have generated much complexity in defining, understanding, and measuring the work-life balance and identifying the consequences of poor work-life balance. Work-life balance cannot be explained with an isolated definition from the above-mentioned but is usually a combination of multiple definitions based on the circumstances of the individuals under study.

Similar to all professions, work-life balance is an essential factor for doctors. Self-care should begin at an undergraduate level. Medical students and young graduates need to balance their time between multiple circumstances including studying, exams, extra-curricular activities, teaching, clinics, and extracurricular activities. The combination of academic overload, stressful and tough exams, and clinical environment can threaten the work-life balance and well-being of medical students and young graduates [7].

Studies have shown the effect of poor work-life balance on the mental and physical health of nurses, doctors, and other front-line workers during the time of the pandemic. Mental and physical health is equally important for all. Similar to long hours of work, mental fatigue by long hours of study can lead to significant problems and should not be ignored. This is to assess the hours spent in study and the hours spent in non-study activities by medical students and graduates in India and to assess the study-life balance among them.

## Materials and methods

The present research employs a cross-sectional observational study design, employing a web-based survey to investigate the study-life balance among medical students and graduates across India. The study was conducted entirely in the virtual realm, with data collection taking place over a period of one month. Ethical clearance was obtained from the Genebandhu Independent Ethics Committee with protocol number ECG001/2023 dated January 17, 2023.

A predesigned questionnaire was designed and made accessible through Google Forms, which was distributed among doctors across India via popular social media platforms. Informed consent was obtained from all participants, providing them with a clear understanding of the research's purpose and reassuring them about the confidentiality of their responses. Furthermore, the questionnaire was also pretested to ensure clarity and relevance, enhancing the validity of the data collected.

The study solely included voluntary participants who meet the inclusion criteria, which are medical students pursuing their MBBS degree and graduates employed by medical institutions in India. Postgraduate students, medical students and graduates who declined participation were excluded from the study. Data management was conducted using Microsoft Excel and data analysis was done using IBM SPSS Statistics Software for Windows, Version 21.0 (IBM Corp., Armonk, NY; Released 2012).

## Results

A total of 416 responses were included in the study. A total of 267 (64.2%) females and 149 (35.8%) males participated in the study. The study participants represent different states of India with Telangana state being the most represented state with 266 participants (63.9%). The participants belong to various academic levels of medical career, with major representation being from the first-year students (140 participants, 33.7%), followed by those in internship (83 participants, 20%).

Table 1 explores the detailed demographic characteristics of the study group including the gender, and the state of India they belong to. Furthermore, it explores the participant's year of study.

**Table 1:** Demographic details of the study participants.

Parameter	N (%)
Gender	
Female	267 (64.2%)
Male	149 (35.8%)
State	
Andhra Pradesh	50 (12%)
Bihar	3 (0.7%)
Gujarat	4 (1%)
Jharkhand	3 (0.7%)
Karnataka	28 (6.7%)
Kerala	4 (1%)
Maharashtra	15 (3.6%)
Odisha	2 (0.5%)
Punjab	6 (1.5%)
Somewhere in India	23 (5.5%)
Tamil Nadu	4 (1%)
Telangana	266 (63.9%)
Tripura	2 (0.5%)
Uttar Pradesh	4 (1%)
West Bengal	2 (0.5%)
Year of Study	
1^st^ year	140 (33.7%)
2^nd^ year	38 (9.1%)
3^rd^ year	54 (13%)
4^th^ year	45 (10.8%)
Intern	83 (20%)
Graduate	56 (13.5%)

Table 2 focuses on the study-life, exploring the place of study, method of studying, hours spent in studying, and also the hours spent in clinical activities which affect their study routine. The use of medical videos in preparation for entrance exams has been widely used in India, this is a key point that was evaluated in this section together with the cumulative stress that comes with studying.

**Table 2:** Analysis of study habits, study environment, and stress levels of students. Values are expressed in N (%).

Parameters	N (%)
Study Place	
Home	321 (77.2)
Library	95 (22.8)
Method of study	
Studying on own	319 (76.7)
Group Study	6 (1.4)
One on one discussion with friend / classmate	91 (21.9)
Hours spent on studies	
None	29 (7)
< 10 hours (1-2 hours/day)	182 (43.8)
10-25 hours (3-4 hrs/day)	113 (27.2)
25-50 hours (5-7 hrs/day)	77 (18.5)
>50 hours ( >8hrs/day)	12 (2.9)
>70 hours ( >10 hrs/day)	3 (0.7)
Hours spent in hospital	
None	145 (34.9)
< 10 hours (1-2 hours/day)	79 (19)
10-25 hours (3-4 hrs/day)	101 (24.3)
25-50 hours (5-7 hrs/day)	42 (10.1)
>50 hours ( >8hrs/day)	32 (7.7)
>70 hours ( >10 hrs/day)	16 (3.9)
Hours spent on watching medical videos	
None	123 (29.6)
< 10 hours (1-2 hours/day)	209 (50.2)
10-25 hours (3-4 hrs/day)	57 (13.7)
25-50 hours (5-7 hrs/day)	18 (4.3)
>50 hours ( >8hrs/day)	6 (1.4)
>70 hours ( >10 hrs/day)	3 (0.7)
Entrance exam	
Within a Year	183 (44)
Within 2 Years	62 (14.9)
After 2 Years	171 (41.1)
Do you feel that your study life is stressful?	
Highly disagree	8 (1.9)
Disagree	4 (1)
Neutral	96 (23.1)
Agree	200 (48.1)
Highly agree	108 (26)

The data from Table 2 reveal that the majority of the participants study at home, 321( 72.2%) while 95 (22.8%) prefer studying in the library. Most respondents, 319 (76.7%) prefer studying on their own, while 91 (21.9%) prefer discussion and only four (1.4%) prefer studying in a group. The number of hours spent in studying and other activities varied among the group. Study shows 182 (43.8%) study < 10 hours a week while three (0.7%) study more than 10 hours a day for studies.

Furthermore, 183 (44%) of the respondents have an entrance exam within a year, 171 (41.1%) after two years, and 62 (14.9%) within two years. In terms of the stress levels related to studies, 108 (26%) responded as highly agreeing to feeling stressed and eight (1.9%) highly disagreed to feeling stressed. This difference was statistically significant (p < 0.001), using the Kruskal-Wallis test.

Various activities that are commonly used by the participants as part of recreation are explored in Table 3. The time spent sleeping, doing household chores and time spent with family is evaluated. Also, time spent by participants in indoor activities like watching entertainment platforms and listening to music is studied. Furthermore, time spent in physical fitness like sports, yoga, and trekking was evaluated alongside leisure activities like arts, hobbies, and long drives. The participants have also quantified the non-educational time spent with friends.

**Table 3:** Activities by the study participants as a part of recreation.

Parameters	N (%)
Time spent at night sleeping every day:	
<6 hours	99 (23.8)
6 - 8 hours	255 (61.3)
8 - 10 hours	54 (13)
>10 hours	8 (1.9)
Do you help with household chores?	
No	196 (47.1)
Yes	220 (52.9)
Time spent in household chores per week:	
None	158 (38)
< 10 hours (1-2 hours/day)	239 (57.5)
10-25 hours (3-4 hrs/day)	19 (4.6)
Do you play outdoor sports/ Yoga/ Fitness activities?	
No	224 (53.8)
Yes	192 (46.2)
Time spent in outdoor sport:	
None	221 (53.1)
< 10 hours (1-2 hours/day)	185 (44.5)
10-25 hours (3-4 hrs/day)	10 (2.4)
Time spent watching television/OTT per week:	
None	45 (10.8)
< 10 hours (1-2 hours/day)	226 (54.5)
10-25 hours (3-4 hrs/day)	115 (27.7)
25-50 hours (5-7 hrs/day)	26 (6.3)
>70 hours (>10 hrs/day)	3 (0.7)
Total	415 (100)
Are you interested in music/art/other hobbies?	
No	58 (14)
Yes	357 (86)
Total	415 (100)
Time spent in music/art/other hobbies per week:	
None	197 (47.4)
< 10 hours (1-2 hours/day)	188 (45.2)
10-25 hours (3-4 hrs/day)	29 (7)
>70 hours (>10 hrs/day)	2 (0.5)
Do you like to go for treks/long drives?	
No	70 (16.8)
Yes	346 (83.2)
Time spent in treks/long drives per week:	
None	339 (81.5)
< 10 hours (1-2 hours/day)	65 (15.6)
10-25 hours (3-4 hrs/day)	6 (1.4)
25-50 hours (5-7 hrs/day)	4 (1)
>50 hours (>8hrs/day)	2 (0.5)
Do you like to go out for dinner?	
No	36 (8.7)
Yes	380 (91.3)
Time spent in going out for dinner per week:	
None	206 (49.5)
< 10 hours (1-2 hours/day)	189 (45.4)
10-25 hours (3-4 hrs/day)	19 (4.6)
25-50 hours (5-7 hrs/day)	2 (0.5)
Time spent with family per week:	
None	135 (32.5)
< 10 hours (1-2 hours/day)	197 (47.4)
10-25 hours (3-4 hrs/day)	71 (17.1)
25-50 hours (5-7 hrs/day)	9 (2.2)
>50 hours (>8hrs/day)	4 (1)
Time spent with friends and colleagues (non-study related) per week:	
None	78 (18.8)
< 10 hours (1-2 hours/day)	220 (52.9)
10-25 hours (3-4 hrs/day)	81 (19.5)
25-50 hours (5-7 hrs/day)	16 (3.8)
>50 hours (>8hrs/day)	19 (4.6)
>70 hours (>10 hrs/day)	2 (0.5)

More than half of the respondents. 255 (61.3%) spend 8-10 hours sleep at night, while 99 (23.8%) sleep < 6 hours. Nearly half of the respondents, 220 (52.9%) admitted to spending their time doing household chores while 196 (47.1%) denied any involvement. About 224 (53.8%) denied engaging in any sport or fitness-related activity.

About 357 (86%) were interested in music and arts, around 197 (47.4%) spent no time on it, while 188 (45.2%) spent less than 10 years on it. Although 346 (83.2%) respondents enjoyed going for treks or long drives, around 339 (81.5%) could not do so. Around 380 (91.3%) enjoyed going out for dinners, 206 (49.5%) never did so. About 197 (47.4%) respondents spent less than 10 hours with family per week while 220 (52.9%) respondents spent less than 10 hours per week with friends and colleagues in non-study-related activities.

**Figure 1:**
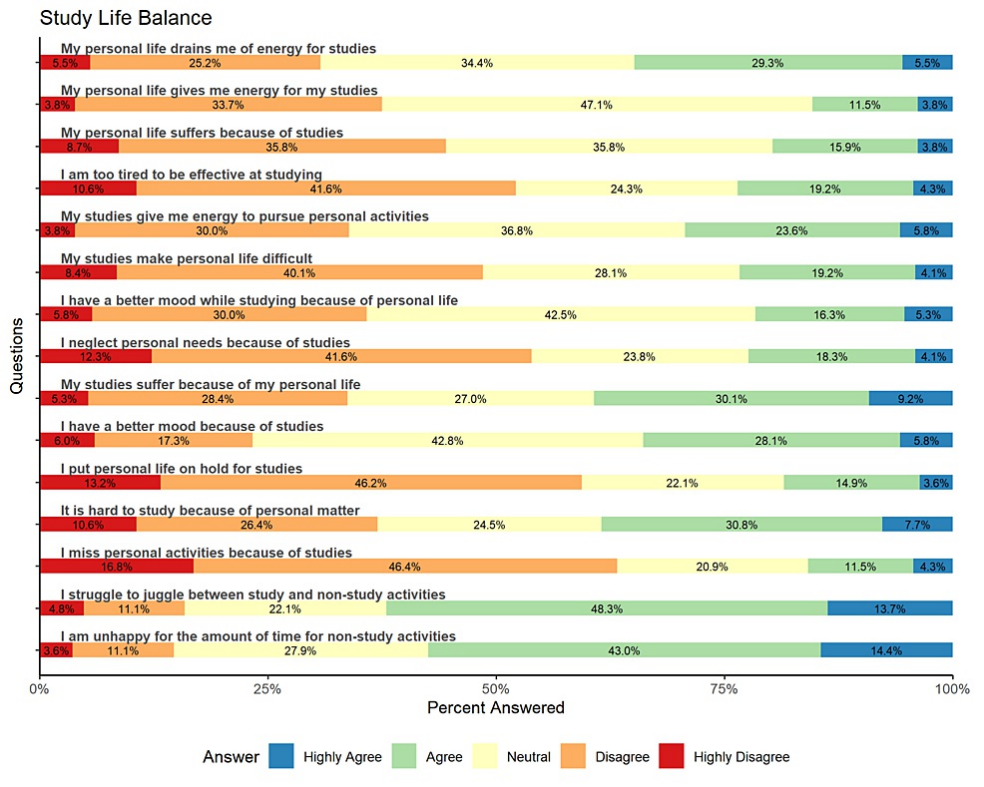
Relationship between studies and personal life of the study participants

The study also tries to explore the relationship between studies and personal life. Figure 1 shows the responses of the participants using the Likert scale to rate the impact of studies on personal life and vice versa.

**Figure 2:**
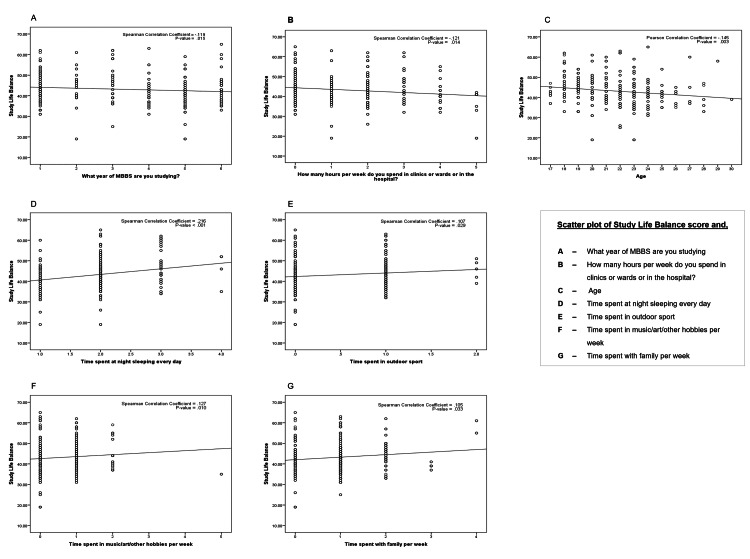
Correlation of study-life balance to appropriate factors

Figure 2 explains the relationship between study-life balance and other variables using the Spearman correlation coefficient. Factors like years of MBBS studies, and time spent in clinical duties showed a weak negative relationship with study-life balance with Spearman correlation coefficient (denoted as ρ) values of -0.119 and -0.121, respectively.

Comparing the study-life balance against factors like time spent in outdoor sport (ρ = 0.107) and time spent with family (ρ = 0.105) showed a weak positive relationship between both variables with very low levels of correlation. Similarly, there is a weak positive relationship between time spent sleeping and study-life balance (ρ = 0.216) as well as time spent on music, art, and other hobbies (ρ = 0.127). There is a weak linear relationship between age and study-life balance as noted by Pearson correlation coefficient of -0.145.

## Discussion

A well-rounded lifestyle that cohesively balances both work and personal life is of utmost importance for the proper functioning of individuals across all sectors, including healthcare. A cross-sectional study by Langade et al. on burnout in medical practitioners reported an alarming level of emotional exhaustion (45% of individuals) and depersonalization (87%) among registered medical practitioners in India [8]. Shanafelt et al. reported that approximately 45% of oncologists in the study sample (n= 1,490), felt emotionally exhausted and depersonalized [9]. To evaluate whether these effects start very early in their healthcare career, our study was designed to evaluate the study-life balance in Indian medical students and graduates. A total of 416 subjects across the various states of India participated in the study, with around 74.1% of the student population finding study-life to be stressful.

Throughout the existing literature, we can find compelling evidence corroborating the findings of our study. Iqbal et al. documented that a majority of medical undergraduate students are affected by depression (51%), stress (53%), and anxiety (67%). Furthermore, morbidity due to stress and anxiety-related causes were greater in fifth-semester students when compared to second-semester students [10]. With regard to sleep quality, Shad et al., by observing and analyzing the Global Pittsburgh Sleep Quality Index (PSQI) have found that the majority of students (62.6%) in general, are poor sleepers, but medical students have poorer quality of sleep (73%) when compared to their non-medical peers (52%) [11].

To battle these dire circumstances and effects of poor work-life or study-life balance, most developed countries, have adapted to a transformation in the medical education system, with a noticeable shift in the direction of prioritizing self-care of students. This implies a departure from the preceding ethos that emphasized majorly on altruism and self-sacrifice [12]. But in many developing countries or less developed countries like India, self-care is yet to be prioritized, which is justified by our study. Our study outlines a clear difference in the time spent on hobbies and studies, indicating burnout. To prevent grave complications, and to restore balance between study and life, Border theory can be implemented for the students in India.

Border theory is a conceptual paradigm that focuses on the “boundaries” an individual establishes between their work and personal life. The theory emphasizes on effective establishment and management of these boundaries by utilizing strategies like segmentation, integration, and boundary management to achieve proper work-life harmony [13]. Medical councils and governing bodies in developing countries such as India, should emphasize the importance of self-care to support students in safeguarding their health and overall well-being amidst the challenges of pursuing a medical degree. This can be done by improving working conditions, giving support for physical and mental health issues, and finally promoting a stable working and studying environment [14]. Organizational support can have a positive influence on maintaining a study-life balance or work-life balance [15]. This support can be given by medical councils and governing bodies of the respective countries.

Strengths and limitations

High response rates, the inclusion of students from diverse institutions across various states, and various academic years are a few of the strengths of our study. However, a few certain limitations are present which include, the impact of institution-specific factors on study-life balance Furthermore, data collection during the exam season might have been influenced by stress, altered study and working patterns, and test-related anxiety. It is possible that few of the responses were an outlet of the above-mentioned stress than facts.

## Conclusions

Our study notes that time spent by medical students was largely for studying and working in hospital. The time spent for recreational activities was less. Self-care and study-life balance is a multi-factorial focal area based on balancing monitoring stress and happiness while completing the tasks of medical school. Extrinsic factors like peer pressure and culture play an important role in this. There was a significant imbalance between the study and life of medical students due to the heavy academic workload with less time spent on leisure activities. Medical students need to receive guidelines for a better transition into medical school. The medical councils and governing bodies also need to regulate self-care guidelines for the better mental health of students.

## Appendices

Appendix _ Study Questionnarie

Title: Study-life balance among medical students

Introduction

You must’ve heard about “WORK-LIFE BALANCE”. Ever heard about “Study-life balance”?

This is a cross-sectional survey to assess study-life balance among medical students and graduates. 

* Indicates required question

I voluntarily agree to participate in this study. *

Yes

No

**Table 4:** Questionnaire

PART 1: DEMOGRAPHICS
1. What is your age? ____________
2. What gender do you identify with?
Male
Female
Others
3. Which state in India do you belong to?
4. What year of MBBS are you studying?
1st year MBBS
2nd year MBBS
3rd year MBBS
4th year MBBS
Intern
Graduate
5. If already graduated please state the year of your graduation.
PART 2 : STUDY LIFE
1. Do you study at:
Library
Home
2. Which method of studying do you prefer:
Studying on own
Group study
One on one discussion with friend / classmate
3. How many hours per week do you spend studying at home or the library?
None
< 10 hours (1-2 hours/day)
10-25 hours (3-4 hrs/day)
25-50 hours (5-7 hrs/day)
>50 hours ( >8hrs/day)
>70 hours ( >10 hrs/day)
4. How many hours per week do you spend in clinics or wards or in the hospital?
None
< 10 hours (1-2 hours/day)
10-25 hours (3-4 hrs/day)
25-50 hours (5-7 hrs/day)
>50 hours ( >8hrs/day)
>70 hours ( >10 hrs/day)
5. How many hours per week do you spend watching medical videos or in the medical coaching classes?
None
< 10 hours (1-2 hours/day)
10-25 hours (3-4 hrs/day)
25-50 hours (5-7 hrs/day)
>50 hours ( >8hrs/day)
>70 hours ( >10 hrs/day)
6. When is your entrance exam?
Within a year
Within 2 years
After 2 years
7. Do you feel that your study life is stressful?
Highly disagree
Disagree
Neutral
Agree
Highly agree
PART 3: NON-STUDY LIFE
1. Time spent at night sleeping every day:
>10 hours
8 - 10 hours
6 - 8 hours
< 6 hours
2. Do you help with household chores?
Yes
No
3. Time spent in household chores per week:
None
< 10 hours (1-2 hrs/day)
10-25 hours(3-4 hrs/day)
25-50 hours(5-7 hrs/day)
>50 hours ( >8 hrs/day)
> 70 (> 10hours/day)
4. Do you play outdoor sports/ Yoga/ Fitness activities?
Yes
No
5. Time spent in outdoor sport:
None
< 10 hours (1-2 hrs/day)
10-25 hours(3-4 hrs/day)
25-50 hours(5-7 hrs/day)
>50 hours ( >8 hrs/day)
> 70 (hours/day)
6. Time spent watching television / OTT per week:
None
< 10 hours (1-2 hrs/day)
10-25 hours(3-4 hrs/day)
25-50 hours(5-7 hrs/day)
>50 hours ( >8 hrs/day)
> 70 (hours/day)
7. Are you interested in music/art/other hobbies?
Yes
No
8. Time spent in music/art/other hobbies per week:
None
< 10 hours (1-2 hrs/day)
10-25 hours(3-4 hrs/day)
25-50 hours(5-7 hrs/day)
>50 hours ( >8 hrs/day)
> 70 (hours/day)
9. Do you like to go for treks/long drives?
Yes
No
10. Time spent in treks/long drives per week:
None
< 10 hours (1-2 hrs/day)
10-25 hours(3-4 hrs/day)
25-50 hours(5-7 hrs/day)
>50 hours ( >8 hrs/day)
> 70 (hours/day)
11. Do you like to go out for dinner?
Yes
No
12. Time spent in it per week:
None
< 10 hours (1-2 hrs/day)
10-25 hours(3-4 hrs/day)
25-50 hours(5-7 hrs/day)
>50 hours ( >8 hrs/day)
> 70 (hours/day)
13. Time spent with family per week:
None
< 10 hours (1-2 hrs/day)
10-25 hours(3-4 hrs/day)
25-50 hours(5-7 hrs/day)
>50 hours ( >8 hrs/day)
> 70 (hours/day)
14. Time spent with friends and colleagues (non-study related) per week
None
< 10 hours (1-2 hrs/day)
10-25 hours(3-4 hrs/day)
25-50 hours(5-7 hrs/day)
>50 hours ( >8 hrs/day)
> 70 (hours/day)
PART 4: CONSTRUCT 1 (STUDY INTERFERES WITH PERSONAL LIFE ACCORDING TO HAYMAN - EXPLAINS STUDY RELATED FACTORS THAT IMPACT AN INDIVIDUAL'S PERSONAL LIFE)
1. My personal life suffers because of studies
Highly disagree
Disagree
Neutral
Agree
Highly agree
2. My studies make personal life difficult
Highly disagree
Disagree
Neutral
Agree
Highly agree
3. I neglect personal needs because of studies
Highly disagree
Disagree
Neutral
Agree
Highly agree
4. I put personal life on hold for studies
Highly disagree
Disagree
Neutral
Agree
Highly agree
5. I miss personal activities because of studies
Highly disagree
Disagree
Neutral
Agree
Highly agree
6. I struggle to juggle between study and non-study activities
Highly disagree
Disagree
Neutral
Agree
Highly agree
7. I am unhappy for the amount of time for non-study activities
Highly disagree
Disagree
Neutral
Agree
Highly agree
PART 4: CONSTRUCT 2 (PERSONAL LIFE INTERFERES WITH STUDY ACCORDING TO HAYMAN - EXPLAINS IMPACT OF OR INTERFERENCE OF ONE'S PERSONAL LIFE ON THEIR STUDIES)
1. My personal life drains me of energy for studies
Highly disagree
Disagree
Neutral
Agree
Highly agree
2. I am too tired to be effective at studying
Highly disagree
Disagree
Neutral
Agree
Highly agree
3. My studies suffer because of my personal life
Highly disagree
Disagree
Neutral
Agree
Highly agree
4. It is hard to study because of personal matter
Highly disagree
Disagree
Neutral
Agree
Highly agree
PART 4: CONSTRUCT 3 (STUDY - PERSONAL LIFE ENHANCEMENT - EXPLAINS HOW STUDIES AND PERSONAL LIFE ENHANCE EACH OTHER. THE ITEMS HELP UNDERSTAND THE SUPPORT AND ENHANCEMENT PROVIDED BY STUDYING ON THEIR PERSONAL LIFE AND VICE VERSA)
1. My personal life gives me energy for my studies
Highly disagree
Disagree
Neutral
Agree
Highly agree
2. My studies give me energy to pursue personal activities
Highly disagree
Disagree
Neutral
Agree
Highly agree
3. I have a better mood while studying because of personal life
Highly disagree
Disagree
Neutral
Agree
Highly agree
4. I have a better mood because of studies
Highly disagree
Disagree
Neutral
Agree
Highly agree
